# A Generative Method for Indoor Localization Using Wi-Fi Fingerprinting

**DOI:** 10.3390/s21072392

**Published:** 2021-03-30

**Authors:** Óscar Belmonte-Fernández, Emilio Sansano-Sansano, Antonio Caballer-Miedes, Raúl Montoliu, Rubén García-Vidal, Arturo Gascó-Compte

**Affiliations:** Institute of New Imaging Technologies, Jaume I University, 12071 Castelló de la Plana, Spain; esansano@uji.es (E.S.-S.); caballer@uji.es (A.C.-M.); montoliu@uji.es (R.M.); vidalr@uji.es (R.G.-V.); agasco@uji.es (A.G.-C.)

**Keywords:** hidden Markov models, indoor localization, machine learning, Wi-Fi fingerprinting

## Abstract

Indoor localization is an enabling technology for pervasive and mobile computing applications. Although different technologies have been proposed for indoor localization, Wi-Fi fingerprinting is one of the most used techniques due to the pervasiveness of Wi-Fi technology. Most Wi-Fi fingerprinting localization methods presented in the literature are discriminative methods. We present a generative method for indoor localization based on Wi-Fi fingerprinting. The Received Signal Strength Indicator received from a Wireless Access Point is modeled by a hidden Markov model. Unlike other algorithms, the use of a hidden Markov model allows ours to take advantage of the temporal autocorrelation present in the Wi-Fi signal. The algorithm estimates the user’s location based on the hidden Markov model, which models the signal and the forward algorithm to determine the likelihood of a given time series of Received Signal Strength Indicators. The proposed method was compared with four other well-known Machine Learning algorithms through extensive experimentation with data collected in real scenarios. The proposed method obtained competitive results in most scenarios tested and was the best method in 17 of 60 experiments performed.

## 1. Introduction

The goal of indoor localization is to estimate the location of a person or object inside a building, where the intensity of the GPS signal is too low to be detected. Indoor localization is an enabling technology for providing services relying on the user’s location information. Some of these services are replicates of GPS-based services, such as route planning, whereas others are specifically new, such us assistive technologies in the realms of Ambient Assisted Living (AAL) and Smart Environments. The acceptance of AAL technologies based on Internet of Things (IoT) for gerontechnology was studied in [1], where societal and economic issues were raised regarding the implementation of IoT for AAL. Remote health care and the well-being of older adults have drawn much attention to indoor localization methods as enabling technologies. User localization was highlighted as a key concept for AAL in [2], in order to provide e-health solutions based on IoT to improve elderly people’s quality of life.

Several technologies have been proposed to address the indoor localization challenge: Bluetooth Low Energy (BLE) [3], sound/ultrasound [4], Wi-Fi fingerprinting [5], video [6], Passive Infrared sensors (PIR) [7], Ultra Wide Band (UWB) [8], magnetic field [9], and Radio Frequency Identification (RFID) [10] are among the most used.

Wi-Fi fingerprinting is one of the most used methods for indoor localization [11]. This is mainly due to the ubiquitous presence of Wi-Fi signals in urban areas, as most mobile phones are equipped with Wi-Fi hardware, and due to its affordable price [12]. Wi-Fi signals can even be used to estimate the best beams to communicate in 5G cellular networks [13].

Different techniques based on Wi-Fi signals have been used for indoor localization [14]: (a) the Wi-Fi signal propagation model uses the sampled intensity of the Wireless Access Points (WAPs) around and a signal path-loss model to estimate the distances between the user and different WAPs; once these distances are estimated, trilateration is used to estimate the user’s location; (b) Time of Arrival (ToA) is based on the time spent by the Wi-Fi signal when traveling between the emitter and the receiver to estimate the distance between them, and then trilateration is used to estimate the user’s location; (c) Angle of Arrival (AoA) uses the times at which the Wi-Fi signal arrives at a set of antennas separated by a known distance to estimate the angle of arrival, and then triangulation can be used to estimate the user’s location; (d) Wi-Fi fingerprinting is based on measuring the Wi-Fi intensity of a signal at different points, creating a so called radio map or Wi-Fi database; then the information in the Wi-Fi database is used to estimate user’s location based on new Wi-Fi samples provided by the user.

Wi-Fi fingerprinting has gained much attention from the research community because it does not need any special hardware, and can achieve accuracies in the range of 0.6 [15] to 1.3 m [16] in controlled scenarios. The information stored in a Wi-Fi radio map presents a high variability, so most Wi-Fi fingerprinting works use Machine Learning (ML) techniques to estimate a user’s location. Machine Learning (ML) techniques are commonly used to detect patterns in the information stored in a Wi-Fi radio map. Then, the uncovered patters are applied on new data provided by the user to estimate his/her location indoors. ML algorithms can be grouped into generative algorithms and discriminative algorithms. Generative ML algorithms are based on Bayes’ theorem, where the posterior probability of the observed features x belonging to class *y*, namely, p(y|x), is calculated using Bayes’ rule:

p(y|x)=p(y)p(x|y)

where the likelihood p(x|y) provides the probability that the observed features x have been generated by the class *y*. Discriminative ML algorithms directly model the mapping from the observed features to the class they belong to, p(y|x). Discriminative ML algorithms are the preferred alternative among the researchers, and the use of generative ML algorithms is less widespread for indoor localization purposes, and when they are used, the temporal autocorrelation present in the Wi-Fi signal is not exploited.

In this work, we present a generative algorithm for indoor localization using Wi-Fi fingerprinting which exploits the temporal autocorrelation present in the Wi-Fi signal. The time series of the Received Signal Strength Indicator (RSSI) received from a WAP is modeled using a hidden Markov model. This allows us to take advantage of the temporal autocorrelation present in the Wi-Fi signal [17], and to estimate the likelihood that another temporal series has been generated by the WAP using the forward algorithm [18]. To the best of our knowledge, this is the first work that models a temporal series of RSSI Wi-Fi samples received from a WAP by means of an HMM, and then uses the forward algorithm on a new temporal series of RSSI Wi-Fi samples to estimate the user’s location.

We compare the performance results of our method in a real scenario with the performance results of other discriminative and generative ML algorithms which do not exploit the temporal autocorrelation present in the data. The comparison shows the competitiveness of the presented method.

The main contributions of this work are:

1. To model the Wi-Fi signal received from a WAP by means of an HMM to preserve the temporal autocorrelation present in real data.
2. To estimate indoor user’s location by means of the forward algorithm for HMM.
3. To compare the performance of the proposed method with the performances of other well-known Machine Learning algorithms used for indoor localization through extensive experiments.

This paper is organized as follows: Section 2 presents previous work on Wi-Fi based indoor localization methods; Section 3 presents background information on modeling Wi-Fi signals and how Machine Learning algorithms are commonly used to estimate an indoor user’s location. Section 4 presents our method to estimate an indoor user’s location which is based on the use of an HMM forward algorithm. Section 5 presents extensive experiments to compare the performance of our method with the performances of other Machine Learning methods commonly used for indoor localization. Section 6 presents the limitations of the proposed method. Finally, Section 7 presents the main conclusions and future lines of research.

## 2. Previous Work

Indoor localization methods can be broadly classified into two main groups: (i) those that need some deployment of infrastructure, as in the case of BLE [4], and (ii) those which do not need the deployment of any infrastructure, as in the case of magnetic field methods [9]. In the same way, those methods which need the deployment of some infrastructure can be subdivided into: (i) those which need ad hoc deployment—for example, systems based on Bluetooth Low Energy (BLE) [9]; and (ii) those which use opportunistic signals from already deployed infrastructures, such as technologies based on Wi-Fi fingerprinting [5].

One of the major advantages of the methods based on WiFi fingerprints is that they make use of already existing WiFi infrastructure. Therefore, the location of the user can be obtained without deploying any additional infrastructures. However, Wi-Fi was not designed for indoor positioning purposes, and so the spread of a radio signal in indoor environments is very hard to predict. In general, this is due to the presence of furniture and walls in the environment which interact with the Wi-Fi signal [19].

Wi-Fi fingerprinting commonly consists of two phases: the offline or training phase, and the online or location estimation phase. During the offline phase, RSSI Wi-Fi signals coming from the surrounding WAPs are sampled at different reference locations within the zone of interest. Then, these data are used to build a model that will be used in the online phase to estimate the user’s location. In the online phase, a location estimation will be provided by the model based on new RSSI Wi-Fi signal sampled by the user [20].

The seminal paper by Bahl et al. [21] presents work on the use of Wi-Fi fingerprinting for indoor localization. The authors first gathered the average signal strength of surrounding WAPs at fixed locations. Then, they compared the resolution of an empirical model and a propagation model. Finally, the authors used the K-Nearest Neighbors (KNN) algorithm in the signal space with the Euclidean distance metrics to estimate the user’s location through triangulation. This work introduces many of the concepts that have been used by other subsequent works, such as the use of a signal propagation model, and the influences of the number of samples taken in the offline phase and the number of visible access points on the quality of the position estimation.

There are few works that have used the temporal autocorrelation present in the RSSI Wi-Fi signal. The seminal paper that mentions the fact that there exists a temporal autocorrelation in the RSSI Wi-Fi signal is [15]. In this work, the RSSI Wi-Fi signal was modeled by means of a Gaussian probability distribution function (pdf), and the value of the autocorrelation for the first lag was used to improve the estimation of the standard deviation for the Gaussian pdf; however, once the Gaussian pdf was modeled, the temporal autocorrelation was not used by the localization algorithm when estimating the user’s location in the online phase.

The generative approach is compared with the discriminative approach in [22]. The authors compare KNN with a Bayesian approximation using two different approaches to estimate the posterior distribution of the user’s location given a set of RSSI samples. In the first approach, the likelihood of the RSSI samples is estimated by a Gaussian kernel method; in the second approach a histogram method is used. Experimental results on a case study show that the generative approach provides better results than KNN. Nevertheless, the generative approach presented in this work does not use the temporal autocorrelation present in the RSSI Wi-Fi signal either in the offline phase or in the online phase.

Another generative approach is presented in [23] based on the use of a Gaussian kernel for indoor localization. After performing spatial filtering on the training dataset, and access point selection to improve location accuracy, the authors estimated the user’s location as a linear function of the points in the Wi-Fi database; the weights of the function were evaluated using a Gaussian kernel. As in the previous case, the temporal autocorrelation present in the RSSI Wi-Fi signal was not used either in the offline phase or in the online phase.

In [24] the authors modeled the RSSI Wi-Fi signal by means of a Gaussian pdf. Several samples were taken by the user, and then they were fitted to a Gaussian pdf. Finally, using Machine Learning algorithms, the parameters of the user’s fitted Gaussian pdf were used to estimate the user’s location. However, although the authors modeled the signal using a Gaussian kernal, the temporal autocorrelation present in the signal was used neither in the offline phase nor in the online phase.

Another generative approach was used in [25] to reduce the computational complexity and thus the energy consumption of mobile devices, during traditional generative fingerprinting indoor localization. Wi-Fi signals were modeled by means of a Gaussian pdf, and some quantities needed to apply Bayes’ theorem were calculated and stored during the training phase to be used in the operational phase. The results showed a saving of about 88% in the number of floating point operations with respect to other algorithms. As in the previous case, the temporal autocorrelation present in the Wi-Fi signal was not used to model the signal.

An ensemble model that fuses Wi-Fi fingerprinting, magnetic field information, and coarse-grained floor plan information is presented in [26]. Hidden Markov models are used to model transitions between rooms in order to improve localization accuracy. Nevertheless, HMM is not used to model RSSI Wi-Fi.

Another ensemble approach for indoor localization based on RSSI Wi-Fi is presented in [27]. The authors utilized the Dempster–Shafer theory of belief functions to calculate the weights of four different classifiers in the decision of the ensemble. Mean and standard deviation were included as features along with RSSI Wi-Fi to improve the classifiers’ accuracies. The experiments showed that the method achieves almost 98% localization accuracy with 2 m localization error. Although the inclusion of mean and standard deviation improved localization, temporal autocorrelation present in the RSSI Wi-Fi signal was not included in the model.

Deep Neural Networks (DNN) have attracted researchers’ attention in recent years due to their great success when applied to complex problems. The authors in [28] used a stacked denoising auto-encoder (SDAE) for indoor localization purposes. To improve the results given by the SDAE, two filters were used: (a) a dynamic Kalman filter to take into account the speed of the user, and (b) an HMM to model transitions between fingerprint points. As in the previous reference, HMM was used to model transitions from one point in the physical space to another, but not to model the RSSI Wi-Fi signal.

Temporal autocorrelation present in the RSSI Wi-Fi signal was also used in [29] in order to reduce the dimensions of a RSSI Wi-Fi fingerprinting matrix database before applying Principal Component Analysis (PCA) to the matrix database. The authors used temporal autocorrelation to reduce redundancy in the matrix before applying PCA in the offline phase. As in the previously presented works, temporal autocorrelation was not used by the localization algorithm when estimating the user’s location in the online phase.

Although temporal autocorrelation was used by some of the previously presented works, it was only used in the online phase, either to better estimate some parameters of the model, as in the case of [15], or to remove redundancy present in the data before building the model in the offline phase, as in the case of [29]. To the best of our knowledge, no previous work used temporal autocorrelation both in the offline phase, to create the indoor localization model, and in the online phase, to estimate the user’s location.

In this work, the Wi-Fi signal received from a WAP is modeled using an HMM (offline phase) which, as presented in Section 4.1, is able to preserve the autocorrelation present in the Wi-Fi signal. Then, given a series of RSSI Wi-Fi samples, the forward algorithm (see Section 3.4) is used to estimate the location of a user (online phase).

## 3. Background

This section introduces the key concepts used in the rest of the paper. First, the two main approaches to model Wi-Fi signals are presented; then, the objective of indoor localization using Wi-Fi fingerprinted is stated; the use of Machine Learning methods for indoor localization is presented in the third subsection; finally, an introduction to HMM is presented.

### 3.1. Wi-Fi Received Signal Strength Indicator Modeling

Two main different approaches have appeared in previous works to model Wi-Fi signal. The first approach describes how the Wi-Fi electromagnetic wave propagates on an environment taking into account multipath and absorption by obstacles present in the environment. The propagation model provides the RSSI of the Wi-Fi signal for every point in the environment. Although this approach is very powerful, it provides a unique value for each point in space instead of a distribution of possible values as in the real case, and thus, this method can not be used to model time series of the RSSI Wi-Fi signal. In [30] the authors presented a Wi-Fi signal propagation model based on the radiosity method. The model uses the geometry of the environment and its transmission and reflection coefficients to solve the illumination equation which in turns provides the RSSI Wi-Fi signal value at each point in the environment. Later, an ad hoc method was used to replicate the temporal autocorrelation present in the signal.

In the second approach, the Wi-Fi signal is individually modeled at each reference point, typically as a Gaussian probability distribution function or a mixture of Gaussian probability distribution functions. Although this approach provides better accuracy for the modeled signal at each reference point considered, values for the RSSI Wi-Fi signal at any other point in the environment should be interpolated.

Autocorrelation in the RSSI Wi-Fi temporal series appeared in [31], and in an improved version in [32] as an important characteristic of the RSSI Wi-Fi signal to be modeled. In addition, the authors showed that RSSI Wi-Fi histograms are, in general, left-skewed, and that a Gaussian fit would not be valid in most cases. Nevertheless, this characteristic was only used by the authors to assess the stability of the signal over time.

The authors in [33], found through experimentation that the RSSI Wi-Fi signal histogram can be modeled by a Gaussian pdf both when there is line of sight (LOS) between the WAP and the user, and also when there is no LOS (non-LOS). Nevertheless, no test was performed to assess the normality of the data distribution.

### 3.2. Wi-Fi Fingerprinting for Indoor Localization

Wi-Fi fingerprinting methods are among the most used indoor localization methods [34]. This is mainly due to the fact that they make use of the Wi-Fi infrastructure already deployed in most urban areas [35]. Among its main drawbacks, one can be find its low accuracy when compared with other indoor localization methods: it is subject to changes in the environment and in the distribution of the WAPs. On the other hand, Wi-Fi fingerprinting accuracy might decay if changes in the environment or a re-distribution on the WAPs locations were performed.

Let $\rho_{w}^{l}\left(t\right)$ be the RSSI sample for WAP *w* at location *l* at time *t*, and let us denote by:

$$R^{l}\left(t\right)=\left\{\rho_{1}^{l}\left(t\right),\rho_{2}^{l}\left(t\right),\ldots ,\rho_{w}^{l}\left(t\right)\right\}$$

the set of RSSI samples for all visible WAPs at a location, which is also called a Wi-Fi fingerprint, l∈[1,L] where *L* is the total number of locations mapped. Commonly, an indoor localization method consists of two phases: offline and online phases. In the offline phase a set of *N* RSSI samples:

$$R^{l}=\left\{R^{l}\left(t_{1}\right),R^{l}\left(t_{2}\right),\ldots ,R^{l}\left(t_{N}\right)\right\}$$

is collected at some reference location *l* between time $t_{1}$ and $t_{N}$. The collection of all RSSI samples for all reference locations:

$$R=\{R^{1},R^{2},\ldots ,R^{L}\}$$

is known as a Wi-Fi fingerprint database, or Wi-Fi radio map. In the online phase, given a new RSSI sample:

$$R\left(t\right)=\{\rho_{1}\left(t\right),\rho_{2}\left(t\right),\ldots ,\rho_{w}\left(t\right)\}$$

the indoor localization method estimates the location $\hat{l}\left(t\right)$ as a function of the information stored in the Wi-Fi fingerprint database:

$$f\left(R\left(t\right)\right)\to \hat{l}\left(t\right)$$

### 3.3. Machine Learning for Indoor Localization

When using ML for indoor localization with Wi-Fi fingerprinting, a model is commonly built and trained using the Wi-Fi fingerprint database collected in the offline phase. Later, given a new Wi-Fi fingerprint, this model is used to estimate the user’s location. Although some ML algorithms only take part in the online phase, like in the case of the K-Nearest Neighbors algorithm (KNN), most of them are trained in the offline phase and later used in the online phase.

A basic technique used to improve the accuracy of the location algorithms is to take more than one sample:

$$\{R\left(t_{1}\right),R\left(t_{2}\right),\ldots ,R\left(t_{S}\right)\},S\in N$$

to estimate the location for each sample:

$$f\left(R\left(t\right)\right)\to \hat{l}\left(t\right),f\left(R\left(t_{1}\right)\right)\to \hat{l}\left(t_{1}\right),\ldots ,f\left(R\left(t_{S}\right)\right)\to \hat{l}\left(t_{S}\right)$$

and to combine the results to provide the final estimate:

$$F\left(\hat{l}\left(t_{1}\right),\hat{l}\left(t_{2}\right),\ldots ,\hat{l}\left(t_{S}\right)\right)\to \hat{l}\left(t_{S},S\right)$$

In [21] results are presented using 1, 2 and 3 samples with the KNN algorithm, and it is stated that the location error diminishes when the number of samples increases.

A Multilayer Perceptron (MLP) is an artificial neural network composed of three layers: an input layer, an output layer, and a hidden layer [36]. The authors of [37] presented an MLP for Wi-Fi fingerprinting indoor localization purposes. The number of neurons in the input layer was equal to the number of WAPs present in the environment, while the number of neurons in the output layer was two, corresponding to a two-dimensional space. The results showed that MLP outperforms probabilistic methods based on maximum likelihood estimation (MLE.)

Naïve Bayes classifiers are probabilistic Machine Learning methods based on Bayes’ theorem. An improved localization algorithm based on naïve Bayes is presented in [38]. The results show that their algorithm outperforms other Machine Learning algorithms such as KNN and J28.

Random Forest (RF) [39] is an ensemble classifier formed by a set of decision trees. It has been extensively used in the Wi-Fi fingerprinting indoor localization realm due to its accuracy and efficiency. In [40] the authors studied RF performance depending on the number of trees used and the depth of the trees. A real application of RF for indoor localization purposes was presented in [41] for locating patients at the room level in a hospital. The authors in [42] compared the RF with other Machine Learning algorithms such as KNN, Weighted KNN (WKNN), and J48, among others, thereby concluding that RF outperforms those algorithms. A study on the accuracy of the algorithm regarding the number of visible WAP used was also presented in this work.

### 3.4. Hidden Markov Models

Following the presentation given in [43], an HMM is characterized by:

1. The number of hidden states *H*. An individual state is denoted as:
  $$S\in \{S_{1},S_{2},\ldots ,S_{H}\}$$
  and the state at time *t* is $q_{t}$.
2. The number of different observation symbols *M*. An individual symbol is denoted as:
  $$V\in \{V_{1},V_{2},\ldots V_{M}\}$$
3. The probability distributions for transitions between two states:
  $$A=\left\{a_{ij}\right\}\;\mathrm{where}:\;a_{ij}=P\left[q_{t+1}=S_{j}|q_{t}=S_{i}\right],\;1\leq i,j\leq H$$
4. The probability distribution for observing a symbol in state *j*:
  $$B=\left\{b_{j}\left(k\right)\right\}\;\mathrm{where}:\;b_{j}\left(k\right)=P\left[v_{k}\;at\;t|q_{t}=S_{j}\right],\;1\leq j\leq N,\;1\leq k\leq M$$
5. The probability distributions for initial states:
  $$\pi_{i}=P\left[q_{1}=S_{i}\right],\;1\leq i\leq H$$

Note that due to characteristic 3, the probability of being at state $S_{j}$ at time t+1 only depends on being at state $S_{i}$ at time *t*, and it is independent of any other previous states $q_{t-1},q_{t-2},\ldots ,q_{1}$. Additionally, note that due to characteristic 4, the probability of observation $b_{j}\left(k\right)$ only depends on states $q_{t}=S_{j}$, and it is independent of any other previous state $q_{t-1},q_{t-2},\ldots ,q_{1}$.

An HMM can be compactly represented as:

λ=(A,B,π)

**Figure 1:**
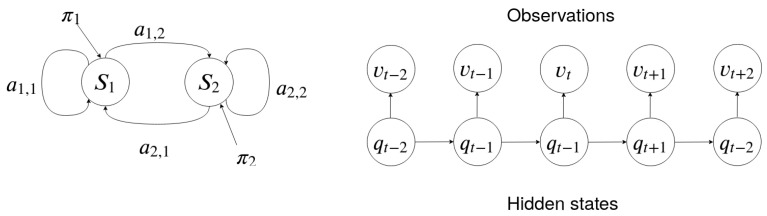
A graphical representation of an HMM with two hidden states on the left. On the right are transitions between a series of states and the observations emitted in each state.

Figure 1 represents an HMM with two states. Each state is represented by a node which contains the probability distribution $b_{j}\left(k\right)$ for observing each symbol:

$$V\in \{V_{1},V_{2},\ldots V_{M}\}$$

Each directed edge represents a transition between two states with probability distribution $a_{ij}$. The arrows represent the probability distribution for the initial states $\pi_{i}$. The observation $O_{t}$ for each state $q_{t}$ and the underlying Markov chain are shown on the right of Figure 1.

An interesting question is (Problem 3 in [43]): for a given number of hidden states *H*, a given number of observation symbols *M*, and a sequence of observations:

$$O=\left\{O_{1},O_{2},\ldots ,O_{T}\right\};\;O_{i}\in V$$

find the HMM denoted by λ=(A,B,π) which maximizes:

$$P\left(O|\lambda \right)=P(\left\{O_{1},O_{2},\ldots ,O_{T}\right\}|\lambda =\left(A,B,\pi \right))$$

One solution to this problem is the Baum–Welch algorithm [18], which is a kind of expectation maximization (EM) method that uses the forward and backward probabilities for estimating *A* and *B* [43]. The forward algorithm in Equation (1) calculates the probability $\alpha_{t}\left(j\right)$ of being at state $q_{t}=S_{j}$ after the sequences of observations $O=\{O_{1},O_{2},\ldots ,O_{t}\}$. This recursive algorithm can be implemented using dynamic programming to efficiently optimize its execution time [44].

(1) $$\begin{array}{cc} \alpha_{1}\left(j\right) & =\pi_{j}b_{j}\left(O_{1}\right);\;1\leq j\leq N\\ \alpha_{t+1}\left(j\right) & =\left[\sum_{i=1}^{H}\alpha_{t}\left(j\right)a_{ij}\right]b_{j}\left(O_{t+1)}\right);\;\left\{\begin{array}{c} 1\leq t\leq T-1\\ \;1\leq j\leq N \end{array}\right.\\ P(O|\lambda ) & =\sum_{j=1}^{H}\alpha_{T}\left(j\right) \end{array}$$

Section 4.2.2 shows how to use the forward algorithm to estimate a user’s location using time series of RSSI Wi-Fi signals.

## 4. Methods

In this section, details are given on how an HMM is used to model the distribution of the time series of RSSI Wi-Fi signals, and how the forward algorithm is used to estimate the user’s location.

### 4.1. Wi-Fi Received Signal Strength Indicator Modeling

Although the Gaussian probability distribution function of RSSI is the most extended way to model a Wi-Fi signal (see Section 2), HMM modeling provides some advantages that are not present in Gaussian modeling, such as better modeling of the histogram of the signal, and most importantly when using a generative model, the autocorrelation in the time series of the RSSI Wi-Fi signal is not lost.

**Figure 2:**
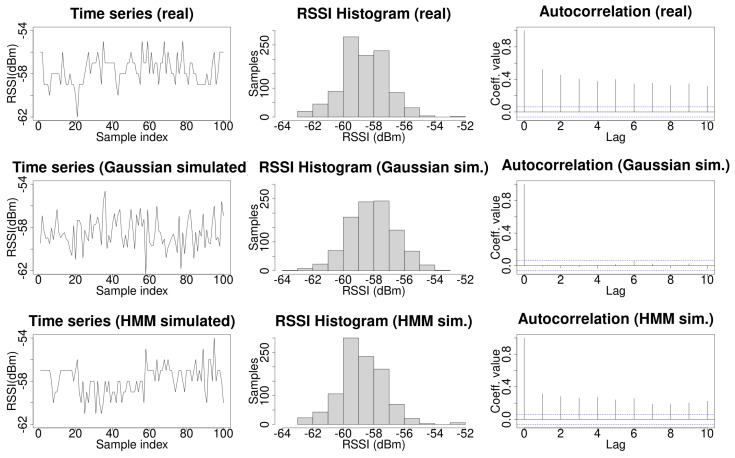
An example of an Received Signal Strength Indicator (RSSI) time series for a Wireless Access Point (WAP) (**left** column), its histogram (**middle** column), and its autocorrelation (**right** column), with real data on the top row, Gaussian simulated data in the middle row, and HMM simulated data along the bottom row.

The top row of Figure 2 shows an example of a real time series of RSSI Wi-Fi signals. The left side plot shows the time series of the Wi-Fi signal. It can be observed that, occasionally, the signal does not change during some consecutive samples. The central plot shows the histogram for 1000 samples. The right plot shows the autocorrelation coefficients which exhibit a strong dependency with the data in the time series. This histogram is typically modeled as a Gaussian probability distribution function. For the particular case of the real data shown in Figure 2, a fit to a Gaussian probability distribution function, using the MASS R library, provides the values μ=−58.15±0.05 and σ=1.55±0.03. By performing a Shapiro–Francia normality test using the DescTools R library, the obtained values were W=0.95574 and $p=4.01\times 10^{-15}$, which allows one to reject the null hypothesis, and to conclude that the data do not follow a normal distribution.The left plot in the middle row of Figure 2 shows a run of 1000 samples generated by the former Gaussian probability distribution function. In this case, a clear difference with the real case can be observed. The values for the simulated signal are independent and change at any new time point in almost all sampled values. For a large number of independent and identically distributed (iid) samples, the autocorrelations of an sequence with finite variance are approximately iid with distribution N(0,1/n) [45]. On the contrary, autocorrelation is present in HMM [17]. The central plot in this row shows the data histogram, in which it can be observed the bell shape of a Gaussian probability distribution function. The right plot shows the autocorrelation coefficients for the data. The autocorrelation present in the real data has totally disappeared in this case. Note that these plots show particular runs along with its histogram and autocorrelation coefficients; another run will generate different data and so a different histogram and autocorrelation coefficients, but for any other examples using a Gaussian probability distribution function, the autocorrelation coefficients will be near zero. A comparison between histograms for real and simulated data using the Kullback–Leibler divergence is presented later in this section.

An HMM can accurately model a time series while preserving autocorrelation when it is used to generate simulated data [17]. The first contribution of this work is to use an HMM to model Wi-Fi signals. The Baum–Welch algorithm [43] was used to fit the time series of the RSSI Wi-Fi signal as an HMM. The number of hidden states for improving the fit of the HMM to the data series can be chosen using the Bayesian Information Criterion (BIC), or the Akaike Information Criterion (AIC), since in our case there is a small difference between the KL divergence in real and simulated data when using 2, 3, or 4 hidden states (as shown in Table 1). Given that the computational time to train an HMM is proportional to the number of hidden states, without loss of generality, an HMM with two hidden states was used to model the HMM in order to minimize training time. The set of initial observation symbols *V* was the different RSSI samples measured for the WAP. The state transition probability distribution $A=\{a_{ij}\}$ was randomly initialized. The observation symbol probability distribution in state $j,\;B=\{b_{j}\left(k\right)\}$ was initialized as the empirical mass distribution function obtained from the real data. Laplacian smoothing was used to deal with the case where no samples were collected for a given RSSI signal intensity, namely, when $b_{j}\left(k\right)=0$. The initial state distribution $\pi_{i}$ was randomly initialized. Fifty iterations were used to build the HMM. The bottom row of Figure 2 shows an example of simulated data generated by the HMM. Note that in this case, as in the real case, the signal does not change during some consecutive samples occasionally. The central plot shows the histogram of the data simulated using an HMM, which is more similar to an actual histogram than in the case of data simulated using a Gaussian probability distribution function. Finally, the right plot shows the autocorrelation coefficients which are not zeros.

A way to compare how close two probability distribution functions are [46] is to use the Kullback–Leibler divergence [47], which measures the difference between two probability functions *P* and *Q* as:

$$D_{KL}\left(P|Q\right)=-\sum_{n}^{}P\left(n\right)\log\frac{P(n)}{Q(n)}$$

In addition, the entropy of a data series H(P), the cross-entropy of two data series H(P,Q) and the Kullback–Leibler divergence are related by the following expressions:

$$\begin{array}{c} H\left(P\right)=-\sum_{n}^{}P\left(n\right)\log P\left(n\right)\\ H\left(P,Q\right)=-\sum_{n}^{}P\left(n\right)\log Q\left(n\right)\\ H\left(P,Q\right)=H\left(P\right)+D_{KL}\left(P|Q\right) \end{array}$$

Let *P* be the empirical mass probability distribution function of the real data, and *Q* the empirical mass probability function from the Gaussian simulated data. A set of 10,000 series containing 1000 elements each from the Gaussian fit were generated using a Gaussian probability distribution function with the previously fitted values μ=−58±0.05 and σ=1.55±0.03, and the Kullback–Leibler divergence was calculated between each of them and the real time series. The same procedure was used to calculate the Kullback–Leibler divergence between real and simulated data generated by an HMM. The results are shown in Table 1. It can be observed that the Kullback–Leibler divergence for the HMM simulated data is lower than for the Gaussian simulated data, so it can be concluded that an HMM fits the real mass probability function better than the Gaussian fit. Additionally, note that the results for the two considered HMMs are very similar regardless of the number of hidden states used to fit the data. Similar results were obtained for the data received from different WAPs.

In light of these results, we propose to use HMM to simulate Wi-Fi data instead of a Gaussian probability distribution function, because it preserves the autocorrelation present in the time series of RSSI Wi-Fi samples received from a WAP, and Kullback–Leibler divergence between the real and simulated data is lower than when using a Gaussian fit.

### 4.2. Location Algorithm

The main difference between the indoor localization method presented in this paper and other methods presented in the literature is that the autocorrelation of the time series of the Wi-Fi signal is used by the location algorithm.

Most indoor localization methods use a set of *S* consecutive samples containing the RSSI received from the *N* surrounding WAPs:

$$\left\{\rho_{1}\left(t_{1}\right),\rho_{2}\left(t_{1}\right),\ldots ,\rho_{N}\left(t_{1}\right)\right\},\left\{\rho_{1}\left(t_{2}\right),\rho_{2}\left(t_{2}\right),\ldots ,\rho_{N}\left(t_{2}\right)\right\},\ldots ,)\left\{\rho_{1}\left(t_{S}\right),\rho_{2}\left(t_{S}\right),\ldots ,\rho_{N}\left(t_{S}\right)\right\}\}$$

Then, each sample in the set is used to estimate user’s location:

$$\begin{array}{ccc} f(\left\{\rho_{1}\left(t_{1}\right),\rho_{2}\left(t_{1}\right),\ldots ,\rho_{N}\left(t_{1}\right)\right)\} & \to & \hat{l}\left(t\right)\\ f(\left\{\rho_{1}\left(t_{2}\right),\rho_{2}\left(t_{2}\right),\ldots ,\rho_{N}\left(t_{2}\right)\right)\} & \to & \hat{l}\left(t_{2}\right)\\ \ldots \\ f(\left\{\rho_{1}\left(t_{S}\right),\rho_{2}\left(t_{S}\right),\ldots ,\rho_{N}\left(t_{S}\right)\right\}) & \to & \hat{l}\left(t_{S}\right) \end{array}$$

**Figure 3:**
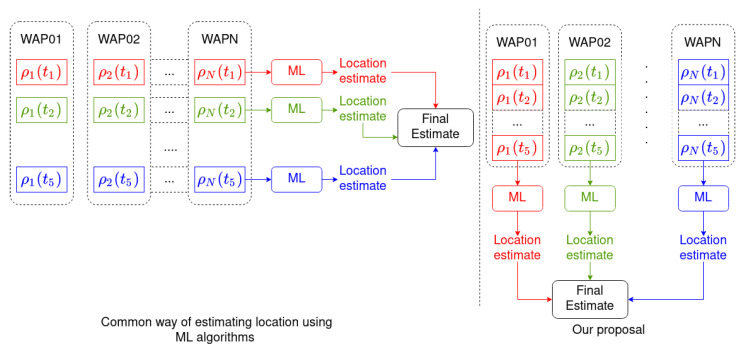
On the left, how most indoor localization methods perform location estimation: a single vector of RSSI samples received from different WAPs is used to estimate the location (horizontal grouping); then several estimations are combined to generate the final estimation. In the method presented in this paper (**right**), a vector of RSSI samples received from the same WAP is used to estimate the location (vertical grouping); then several estimations are combined to generate the final estimation. A sample size of 5 is given as an example. Colors have been used to show the different paths through which the data are processed in both approximations.

Afterwards, the results are combined to provide a final location estimation [21] (horizontal grouping with same color in the left hand side of Figure 3, with S=5 as an example):

$$F\left(\hat{l}\left(t_{1}\right),\hat{l}\left(t_{2}\right),\ldots ,\hat{l}\left(t_{S}\right)\right)\to \hat{l}\left(t_{S},S\right);$$

temporal correlation in the RSSI Wi-Fi signal is not used by these methods.

In this work, the time series of *S* RSSI Wi-Fi samples received from each one of the *N* surrounding WAPs,

$$\left\{\rho_{n}\left(t_{1}\right),\rho_{n}\left(t_{2}\right),\ldots ,\rho_{n}\left(t_{S}\right)\right\}\;\mathrm{with}\;n\in \left\{1..N\right\},$$

is used to estimate user’s location (vertical grouping with the same color as the right hand side of Figure 3):

$$f\left(\left\{\rho_{n}\left(t_{1}\right),\rho_{n}\left(t_{2}\right),\ldots ,\rho_{n}\left(t_{S}\right)\right\}\right)\to {\hat{l}}_{n}\left(t_{S},S\right)$$

This procedure is repeated for each one of the *N* WAPs present in the environment, providing *N* location estimates:

$$\begin{array}{ccc} f(\left\{\rho_{1}\left(t_{1}\right),\rho_{1}\left(t_{2}\right),\ldots ,\rho_{1}\left(t_{S}\right)\right\}) & \to & {\hat{l}}_{1}\left(t_{S},S\right)\\ f(\left\{\rho_{2}\left(t_{1}\right),\rho_{2}\left(t_{2}\right),\ldots ,\rho_{2}\left(t_{S}\right)\right\}) & \to & {\hat{l}}_{2}\left(t_{S},S\right)\\ \ldots \\ f(\left\{\rho_{N}\left(t_{1}\right),\rho_{N}\left(t_{2}\right),\ldots ,\rho_{N}\left(t_{S}\right)\right\}) & \to & {\hat{l}}_{N}\left(t_{S},S\right) \end{array}$$

Afterwards the *N* location estimates are combined to provide the final location estimate:

$$F\left({\hat{l}}_{1}\left(t,S\right),{\hat{l}}_{2}\left(t,S\right),\ldots ,{\hat{l}}_{N}\left(t,S\right)\right)\to \hat{l}\left(t_{S},S\right)$$

The advantage of this approach is that the temporal autocorrelation present in the RSSI Wi-Fi signal is used by the location algorithm, namely, the autocorrelation in the time series shown in Section 4.1. Figure 3 depicts how our location algorithm works and compares it with other Machine Learning algorithms for indoor localization. Note that in Figure 3 colors have been used to group the data that are processed together in both approximations.

Another important difference from other algorithms is that no metrics are used to measure the distances between Wi-Fi samples. In fact, the symbols used to represent the RSSI samples, typically measured in dBm (for example, the time series −40, −41, −42, −42, −41) could be replaced by any other set of symbols (for example, a,b,c,c,b, where the symbol −40 has been replaced by *a*, −41 by *b*, and −42 by *c*). This avoids the need fir giving an ad hoc value when a reading for an RSSI is absent. For example, when using the KNN algorithm and the Euclidean distance metrics, and there is no signal measured for a particular WAP, usually it is substituted by the ad hoc value −100 dBm, which could have an impact when measuring the distances from other samples in the Wi-Fi database. In this work, no distance between samples is used; instead, the autocorrelation present in the time series of symbols is used.

#### 4.2.1. Offline Phase

**Figure 4:**
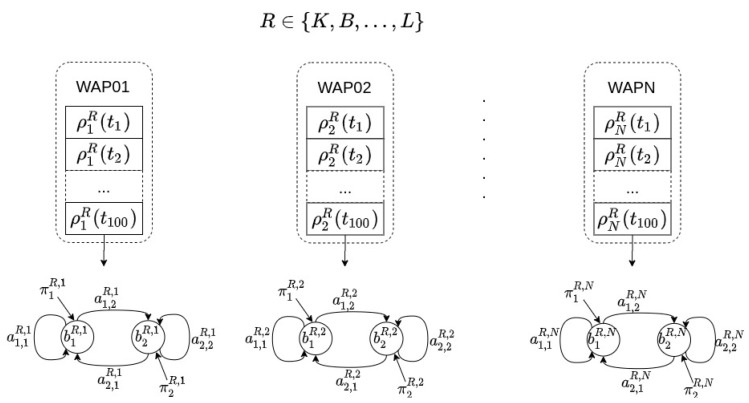
Offline phase. For each room (*R*) and for each time series received from a WAP present in the Wi-Fi fingerprint database, an HMM was built. Different rooms will have different parameters in their HMMs for the same WAP. Common rooms in an apartment are used in this particular example: K,B, and *L* stand for kitchen, bedroom, and living room.

After collecting the Wi-Fi fingerprint database (details are given in Section 5.1), a model is built in order to locate a user at room level in the following way: for each room in the environment (*R*), and for each WAP present in the database (*N*), an HMM is built, namely, each room *R* is modeled by *N* different HMMs. The total number of HMMs in any location model is R×N (see Figure 4).

As an example, let us assume that a user has collected Wi-Fi fingerprint samples in an apartment composed of three different rooms: a bedroom, a kitchen, and a living room. Moreover, let us also assume that 10 different WAPs were seen in whole environment. With the previous assumptions, the user takes 100 Wi-Fi training samples in each room. Afterwords, for each room in the apartment and for each WAP present in the environment, our method builds an HMM using the set of Wi-Fi training samples. Namely, each room has 10 HMMs associated with it for indoor localization purposes. Figure 4 shows each of the three rooms (bedroom, kitchen, and living room) with their associated HMMs.

An issue could arise when comparing the location estimates given by two different HMMs. To better understand this issue, let us consider the following example. On the one hand, let us assume a WAP which is barely seen for a given location. An example of an RSSI time series of length 100 samples for such a WAP might be [−100, −100, −90, −100,..., −100] where suspension points represent the value −100, namely, most of the data in the series are −100, meaning no sample was collected. Once an HMM was built with this time series, a sequence containing only −100 values will be assigned with a high probability. Obviously, this means that the WAP can be barely seen from that location. In terms of information gain, a sequence of five samples [−100, −100, −100, −100, −100] has a high probability but provides low information gain. Note that this result is equally true for any other room from which the WAP can be barely seen. On the other hand, let us assume the example in Figure 2 which might not provide a probability for the estimated location as high as the former HMM, even in the case that the WAP has been effectively set in the estimated room. This is due to the fact that the number of possible trails when building the HMM is higher in this case than in the case in which the WAP is barely seen. In terms of information gain, although a trail of length five might have a lower probability than in the previous sequence having the −100 value for all samples in the trail, the information gain provided by it could be higher than in the previous case. Thus, to be able to compare both estimates, the strategy is to normalize the estimate to the maximum probability of the data series used when building the HMM. To calculate the maximum probability, the number of RSSI Wi-Fi samples should be previously chosen. Let us denote this number as *s*, and this will be the number of Wi-Fi samples used by the location algorithm in the online phase. Then, the maximum probability is obtained by means of the forward algorithm applied to sequences of size *s* on the training data. This maximum probability depends on the sample size *s* and should be re-calculated if *s* changes, but this does not mean the HMM has to be retrained again. Instead, the forward algorithm can be used to find out the maximum for the new sample size.

Once all HMMs have been built, the location algorithm will be ready to estimate the user’s location in the online phase.

#### 4.2.2. Online Phase

In the online phase, the user’s device receives a time series of *S* samples. Each sample contains the RSSI of the surrounding WAPs. If there is no RSSI for a particular WAP in the database, the value −100 is used instead. Thus, a time series of *S* RSSI samples is received for each of the *N* WAPs present in the database:

(2) $$\left\{\rho_{n}\left(t_{1}\right),\rho_{n}\left(t_{2}\right),\ldots ,\rho_{n}\left(t_{S}\right)\right\},n\in \left[1..N\right]$$

Then, for each room *R* in the environment (see Figure 4) and for each HMM associated with the room, the forward algorithm is used to calculate the probability that the time series has been generate by each HMM at the room. The final probability associated with the room is the product of the probabilities for each HMM:

(3) $$p\left(R\right)=\prod_{n=1}^{N}forward_{R}\left(\rho_{n}\left(t_{1}\right),\rho_{n}\left(t_{2}\right),\ldots ,\rho_{n}\left(t_{S}\right)\right)$$

where *R* is each of the rooms present in the environment, i.e., R∈{Kitchen,Bedroom,Livingroom}.

Finally, the room estimated is the one that provides the maximum probability in Equation (3):

(4) $$\hat{R}=argmax_{R\in \{K,B,\ldots ,L\}}\prod_{n=1}^{N}forward_{R}\left(\rho_{n}\left(t_{1}\right),\rho_{n}\left(t_{2}\right),\ldots ,\rho_{n}\left(t_{S}\right)\right)$$

**Figure 5:**
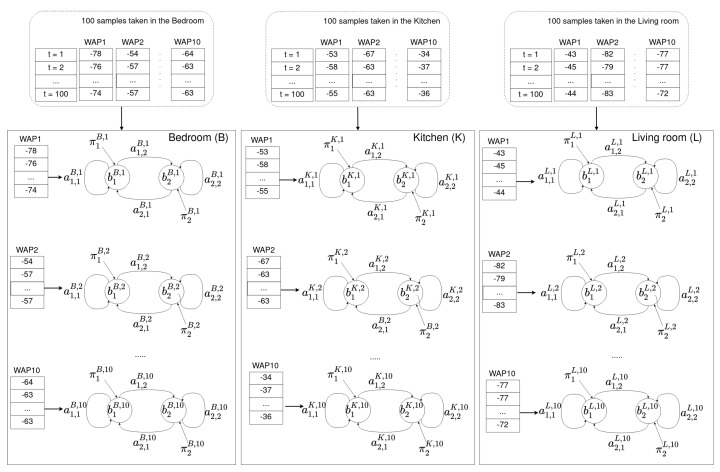
An example of an offline phase. For each room (bedroom, kitchen, and living room), and for each time series received from a WAP present in the environment (10 in this example), an HMM was built. Note that the set of training samples is different for the three locations: bedroom, kitchen, and living room.

**Figure 6:**
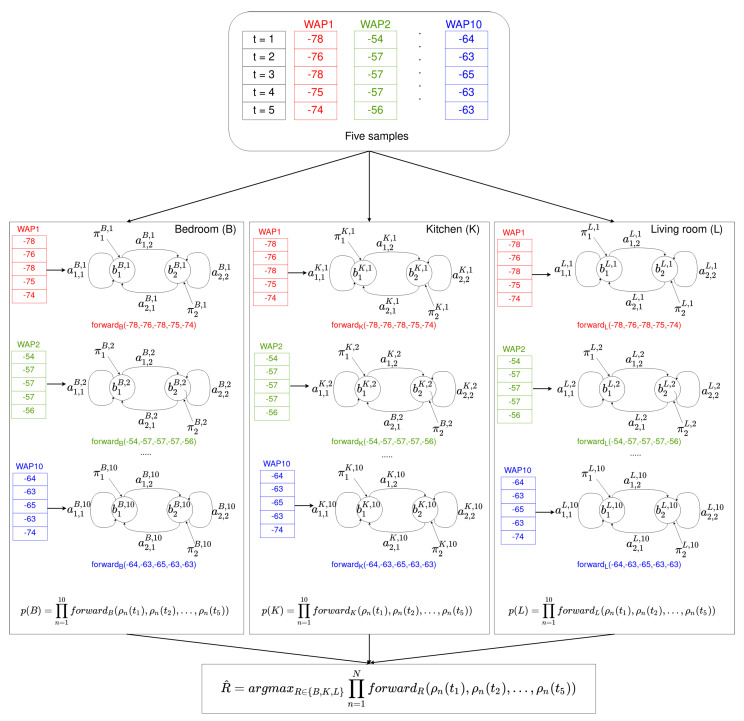
An example of the offline phase. For each room (bedroom, kitchen, and living room) and for each time series received from a WAP present in the environment (10 in this example), an HMM was built. Note that for each sequence of 5 samples arriving from a WAP (same color), each of the 10 HMMs provides a probability using the forward algorithm. The probability of being at rooms {bedroom, kitchen, living room} is evaluated as the product of the 10 previous probabilities. Finally, the estimated room $\hat{R}$ is such that it provides the maximum product.

This example uses the HMM shown in Figure 5. An example of how the online phase performs is shown in Figure 6.

It could happen that the probability for a new data series would be greater than the maximum probability of the data series used when building the HMM (Section 4.2.1), in which case the maximum probability is updated with the new value.

To analyze the computational cost it is useful to write Equation (1) in its matrix form as in Equation (5), for one WAP.

(5) $$forward\left(\rho \left(t_{1}\right),\rho \left(t_{2}\right),\ldots ,\rho \left(t_{S}\right)\right)=\prod_{n=1}^{S}\pi^{t}A^{n}\rho \left(t_{n}\right)$$

where $\pi^{t}$ is the transpose vector of initial probability distributions for the *H* states in the HMM, and *A* is the matrix containing the probability distributions for two states: $A=\{a_{ij}\}$, with {i,j}∈{1,H}.

$$A=\left\{a_{ij}\right\},\;\mathrm{with}\;\left\{i,j\right\}\in \left\{1,H\right\}$$

Each vector $\pi^{t}A^{n}$ has one row and *H* columns and can be pre-computed and stored for later use, so each factor in Equation (5) consists of *H* multiplications and H−1 additions. If the number of samples in the time series is *S*, the number of WAPs in the database is *W*, and the number of rooms is *R*, the total number of multiplications to classify a time series is S×W×R×H and the total number of additions is S×W×R×(H−1).

## 5. Experiments and Results

This section first presents how data for experimentation was collected and prepared; afterwards, a comparison of the presented method and other well-known ML methods is presented.

### 5.1. Data Acquisition and Preparation

Data for experimentation were collected in three different environments by three volunteers. The three environments were urban flats. All data were collected by means of the application presented in [11]. The three volunteers used the same device model to collect the data, a Sony Smartwatch 3 (4 GB, Quad-Core 1.2 GHz, 512 MB RAM), running WearOS. This same device model was also used by the volunteers in the online phase. Each volunteer chose the rooms to be tracked at. The first volunteer (male, 49 years old) chose six rooms, the second volunteer (male, 30 years old) chose five rooms, and the third volunteer (female, 26 years old) chose four rooms.

The aim of these experiments was to be as realistic as possible, so they were carried out in real environments. It was assumed that each user would collect the training and tests datasets on different days and at their own pace. It was also assumed that different users would choose different rooms to be mapped. The only common point was that all users collected the same number of samples (100 samples) in each selected room for the training dataset. Wi-Fi fingerprint training databases were collected by the users in three different ways. We have experimentally found that taking 100 samples is a good compromise between the time it takes to sample them and the performance of the algorithm in the online phase. In the first way, each user took 100 Wi-Fi samples standing up in the center of each room. In the second way, the user took 100 Wi-Fi samples while walking randomly around the room. In the third way, 100 samples were taken by the user at his/her most common location in the room; in this last case the actual position of the user was unknown—he/she could be laying on the sofa in front of the TV set, or sitting in a chair. The users reported that they did not change their common locations during the experimental phase. Therefore, 300 Wi-Fi samples were collected by each user in each room. All users reported taking about two minutes to collect 100 samples in each room, so on average each sample was taken in just over a second. There was no temporal restriction on the procedure to collect the training data by the users; some of them decided to map all rooms on the same day, while others mapped different rooms in different days.

After the training database was collected, each user collected data for the test dataset during two weeks. During this period, each user wore the smart watch all day, even in his/her own home, and it was charged at night. A single charge was enough in all cases to keep the smart watch running the sampling application as a background process. Every minute, five consecutive measures were taken by the smart watch and sent to the server where all data were collected. In order to know the real locations, and to avoid disturbing the users with an exhaustive procedure, they were asked to register the real locations at their own discretion. Finally, only measures from the test dataset where users registered their locations were used in the testing phase. The total number of WAPs detected at each flat, the total number of samples, and the number of samples at each room for testing purposes are shown in Table 2. Note that the percentages of samples in each room are different; there are more samples from rooms where the users spent more time. This fact did not bias the experimental results because the experiments tried to compare the performances of different ML algorithms using the same Wi-Fi databases. Moreover, these databases reflect the real behavior of the users in their own homes. Finally, note that user 2 did not include the living room in the experiment. In the same way, user 3 did not include either the living room or the bathroom in the experiment.

### 5.2. Performance Comparison

The aim of this section is to show the validity of the proposed Wi-Fi fingerprinting location algorithm rather than selecting the optimal closest set of parameters for each algorithm.

Our location algorithm has been compared with other four well-known ML algorithms: K-Nearest Neighbors (KNN), Random Forest (RF), Naïve Bayes (NB), and Multi-Layer Perceptron (MLP). KNN is one of the most used Machine Learning algorithms for indoor localization purposes. The algorithms (KNN, RF, NB and MLP) with which we have compared our method (HMM) are among the most commonly used for Wi-Fi indoor localization purposes [48]. Although different distance metrics can be applied using this algorithm the most extensively metrics used is the Euclidean one. The number of neighbors used was K=1. RF is based on an ensemble of decision trees and is a general purpose ML algorithm which provides high precision on most different applications. All attributes were used to randomly investigate when creating the forest, and the number of iterations used to build the model was 100 in all cases. MLP is a neural-network algorithm, the learning rate was set to 0.3, the momentum rate for back propagation was set to 0.2, the number of epochs was set to 500, the number of hidden layers was half the sum of attributes and classes. NB is a generative algorithm based on Bayesian Statistics, it has no parameters to fix. We have used the implementation provided by Weka [49] for these algorithms. In all cases, the room estimated was that which provided the maximum accumulated probability as an addition of the probabilities for all consecutive samples used. Experimentally we tested that this strategy provided better results than a voting strategy.

**Figure 7:**
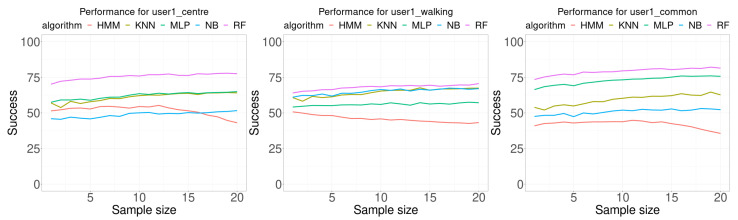
Performance comparison for user 1. Each curve plots the successful rate (percentage) as a function of the number of consecutive samples used for classification (sample size). Each Wi-Fi database contained 100 samples per room for building the model.

Figure 7 shows the results of the experiments performed using the data for user 1. Each plot in this Figure shows the accuracy, as the percentage of correctly classified samples, as function of the number of samples used in the classification (sample size). Each Wi-Fi database contains 100 samples per room for building the model. The training data for the first plot was collected by the user in three different ways: standing at the center of the room, randomly walking around the room, and standing at the most common location in the room. The maximum sample size used was 20, this allows us to check the performance trend as the number of samples increases. It should also be noted that most of the methods presented in the literature use a single sample.

In general, it is expected than the higher the number of samples used for classification, the best accuracy results would be obtained. This assumption is generally true for all algorithms tested except for the HMM algorithm. All algorithms, except HMM, show an ascending trend as the number of samples for classification increases. The HMM classification algorithm presents a maximum near the central value of the sample size when the data was collected at the center of the room or at the most common location of the user, but decreases as the number of sample size increases when the training samples were collected for the user randomly walking around the room.

Table 3 presents the summary of data shown in Figure 7. The first row (min) is the minimum in the percentage of the accuracy for all sample sizes tested. The second row (size.min) is the sample size where the minimum happened. The third row (max) is the maximum in the percentage of the accuracy for all sample sizes tested. The fourth row (size.max) is the sample size where the maximum happened. The fifth row (avg) is the average of the accuracy for all sample sizes. The sixth row (diff) is the average of the difference between the best accuracy percentage and the percentage of the algorithm at the corresponding column. The last row (best) is the number of times the algorithm provided the best accuracy. All algorithms, except HMM, provided the minimum of the accuracy at a smaller sample size than for the maximum value of the accuracy. For all performed experiments with data for user 1, RF algorithm provides the best results for any sample size.

**Figure 8:**
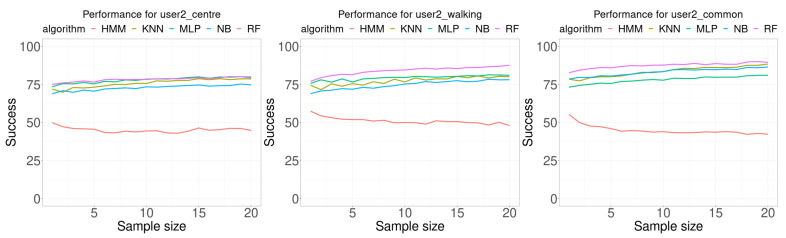
Performance comparison for user 2. Each curve plots the successful rate (percentage) as a function of the number of consecutive samples used for classification (sample size). Each Wi-Fi database contained 100 samples per room for building the model.

Figure 8 shows the results of the experiments performed using the data for user 2, and Table 4 presents a summary of the results. Results are very similar to those for user 1. For all classification algorithms, except HMM, as the sample size increase the accuracy also increases. The first difference is regarding the algorithm that provided the best results, which is MLP on 8 of 20 times for the test database collected by the user at the center of the room, and 12 of 20 times for RF.

**Figure 9:**
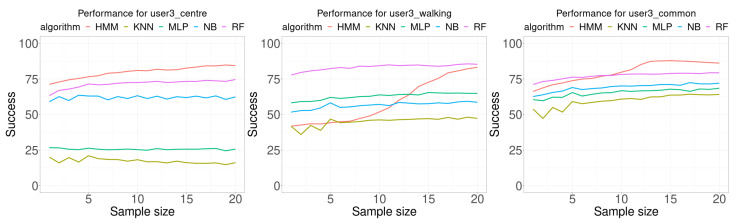
Performance comparison for user 3. Each curve plots the successful rate (percentage) as a function of the number of consecutive samples used for classification (sample size). Each Wi-Fi database contained 100 samples per room for building the model.

Figure 9 shows the results for user 3. This case is very different from the two previous cases. For all classification algorithms, as the sample size increases the accuracy increases also. In addition, there is a remarkable result for the random case, which presents an S-shape for the HMM algorithm. Table 5 presents a summary of the results for user 3. There are two noticeable results, first the HMM classification algorithm provided the best results for any sample size for the training database collected in the center of the room (20 of 20), and it provided the best results in 12 of 20 sample sizes for the training data collected at the “most usual” location used by the user.

**Figure 10:**
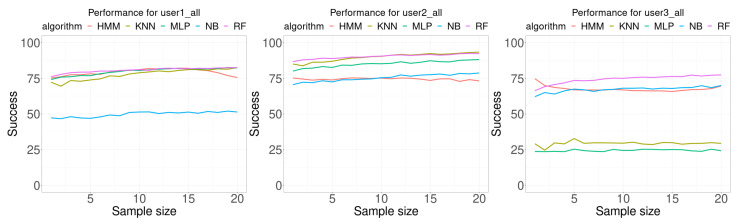
Performance comparison. Each curve plots the successful rate (percentage) as a function of the number of consecutive samples used for classification (sample size). Each Wi-Fi database contained 300 samples per room for building the model (100 at the center of the room, 100 from random walking, and 100 in the most common location in the room).

In the last experiments, the three training datasets (center, random, and common) were combined to build each classifier. Figure 10 shows the performance results for these classifiers, and Table 6 shows a summary of the results. In all cases, except for NB in user 1 and user 2 and RF in user 3, the average accuracy, the minimum accuracy and the maximum accuracy increased or was the same. For user 1, MLP provided the best result in 1 case, HMM in 5 cases and RF in 14. For user 2, KNN provided the best result in 10 cases and RF in 10 cases. For user 3, RF provided the best result in 8 cases and HMM in 12 classes. In summary, MLP provided the best result in 1 case, KNN in 10 cases, HMM in 17 cases and RF in 32 cases. The only algorithm that never reported a best case was NB.

The results of the experiments show that the presented algorithm (HMM), based on the use of HMM for indoor localization, provided an accuracy close to the other algorithms it was compared with.

**Figure 11:**
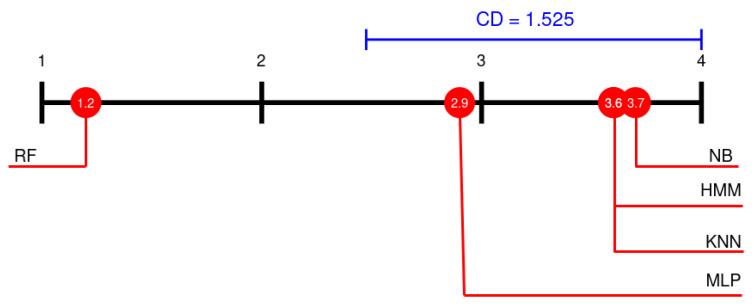
Comparison of the performances of methods using the Nemenyi test. CD is critical distance.

In order to compare the mean rank of the classification algorithm chosen on the 12 datasets used, Friedman and Nemenyi tests were performed. The result for Friedman test was $\chi^{2}$ = 21.6 and *p*-value = 0.0002407, so we reject the hypothesis that all the algorithms are equivalent [50]. To compare the results provided by the classification algorithm chosen, a Nemenyi’s test was performed [51]. Results for the Nemenyi’s test are presented in Figure 11 using the Demsra diagram. These results show that the differences between the proposed method (HMM) and the methods NB, KNN and MLP are less than the critical distance (CD), so this differences are no statistically significant. Moreover, we also performed the z-test, at 95% significant level (see Table 7). These results show that the performance of the RF algorithm is significant better than the rest algorithms. On the contrary the performance of the MLP, HMM, KNN and NB algorithms is not significant different under the critical distance.

The database user2_centre and its corresponding test database were chosen to compare the computational cost between the algorithms used. All time series of five consecutive samples present in the test database were classified by each algorithm, and the time was tracked. This experiment was repeated 100 times for each algorithm. Table 8 presents the results for the mean time and the mean squared error for classifying each time series. It is worth to mention that the implementation of the forward algorithm is not optimised, the computational cost could be reduced using the implementation strategy given in Section 4.2.2. Experiments were performed using a computer equipped with an i7-8750 CPU, 16 GB of RAM, running Ubuntu 19.10. The code was developed using the Java programming language (OpenJDK 14).

## 6. Discussion

The presented method for indoor localization assumes that RSSI Wi-Fi signals are autocorrelated. This characteristic of the Wi-Fi signal allows one to model it by means of an HMM. Although we have check this point experimentally, and the results were always compatible with this assumption, it could be scenarios where this could not be the case. Thus, this assumption should be checked before using the presented method for indoor localization.

As stated in Section 5.2 the aim of this work is to show the validity of the proposed Wi-Fi fingerprinting indoor localization algorithm rather than selecting its optimal closest set of parameters for each scenario. Nevertheless, the impact of the selected parameters, such as the number of RSSI Wi-Fi samples in the training set and the number of hidden states, might be studied to get a proper view of the impact of these parameters in the accuracy of the method.

Another issue, present in all Wi-Fi fingerprinting indoor localization methods, that needs to be studied in more detail, is the decay in the accuracy when environmental changes happen, for example physical changes in WAPs location or in the furniture.

Although the aim of the experimental phase was to be as much realistic as possible, user’s behavior during the experimental phase could have biased the results in some scenarios. In order to detect these bias, it would be useful to perform an experiment where two people share the same urban flat but with different behaviours.

## 7. Conclusions

A new method for indoor localization based on Wi-Fi fingerprinting was presented. The method is based on modeling the RSSI Wi-Fi signal by means of an HMM, which preserves the autocorrelation present in the Wi-Fi signal. The estimation of the user’s location is performed using the probability of a time series of samples provided by the forward algorithm.

Extensive experimentation was performed on three real scenarios (urban flats). The results showed that the performance of the presented method dramatically improved with the number of samples used in the training stage. The accuracy performance of the presented method was compared with other well-known ML methods commonly used in indoor localization. The results showed that the presented method was the second best method (it provided 17 times the best results over 60 experiments; see Table 6), only behind the Random Forest algorithm (it provided 32 times the best results over 60 experiments; see Table 6). The results showed that the presented method was competitive when compared with other well know Machine Learning methods used for indoor localization.

In one scenario, the performance decayed down to twenty five iterations, and then it started to increase providing a performance for fifty iterations similar than to the performance for fifteen iterations.

Nowadays, there exists strong evidence on the associations of circadian rhythm anomalies and neurodegenerative diseases [52], particularly between mobility patterns in-home [53] or during outdoor time [54] and cognitive decay. Changes in these patters in-home could indicate the onset of a cognitive impairment [55], or the onset of a contagious disease [56]. We are planning to use the presented algorithm to monitor older adults living alone in order to early detect cognitive decay.

## Figures and Tables

**Table 1:** Entropy H(P), Kullback–Leibler divergence $D_{KL}\left(P|Q\right)$, and cross-entropy H(P,Q) for the empirical probability distribution functions of real data *P* and simulated data generated with a Gaussian probability distribution function and an HMM model with 2, 3, and 4 states *Q*. Fifty iterations were performed to build the HMM models; 10,000 experiments with 1000 samples each were done.

	Entropy	
Real data	2.633	
	KL-divergence	Cross-Entropy
Gaussian simulated	0.121 ± 0.018	2.752 ± 0.018
HMM (2 states) simulated	0.023 ± 0.001	2.655 ± 0.024
HMM (3 states) simulated	0.023 ± 0.001	2.656 ± 0.022
HMM (4 states) simulated	0.024 ± 0.001	2.658 ± 0.024

**Table 2:** Total number of different WAPs detected at each environment, and the number of samples taken in different rooms.

Environment	Number of	Total	Test Samples at					
Code Name	WAPs	Test Samples	Bathroom	Kitchen	Dining Room	Office	Bedroom	Living Room
User1	34	17,410	480 (2.76%)	995 (5.76%)	1261 (7.23%)	1214 (6.93%)	6570 (37.72%)	6890 (39.57%)
User2	74	17,953	325 (1.81%)	907 (5.05%)	1493 (8.32%)	7709 (42.94%)	7519 (41.88%)	-
User3	88	17,022	-	360 (2.11%)	1035 (6.08%)	14202 (83.43%)	1425 (8.37%)	-

**Table 3:** Summary of the data shown in Figure 7. The rows for minimum (min.), maximum (max.), average (avg.), and difference (diff.) are given in percentages of successful classification experiments. The row labeled as size is for the corresponding sample size. The row labeled as #best is for the number of times the corresponding algorithm provided the best results.

	User1_Centre					User1_Walking					User1_Common				
	HMM	KNN	RF	NB	MLP	HMM	KNN	RF	NB	MLP	HMM	KNN	RF	NB	MLP
min. (%)	43.09	53.77	70.20	45.51	57.50	42.61	58.24	64.01	61.05	54.14	35.60	52.03	73.46	47.37	66.39
sample size	20	2	1	2	1	19	2	1	1	1	20	2	1	5	1
max. (%)	55.28	64.29	77.88	51.61	64.98	50.74	67.63	70.62	67.42	57.50	44.81	64.62	82.15	53.06	76.01
sample size	12	19	19	20	20	1	14	20	17	19	11	19	19	18	19
avg. (%)	51.74	61.02	75.61	48.76	62.13	45.76	64.36	68.07	65.01	56.01	42.14	59.33	79.16	50.82	72.76
diff. (%)	23.87	14.59	0.00	26.85	13.49	22.31	3.71	0.00	3.06	12.05	37.03	19.84	0.00	28.34	6.41
#best	0	0	20	0	0	0	0	20	0	0	0	0	20	0	0

**Table 4:** Summary of the data shown in Figure 8. The rows for minimum (min.), maximum (max.), average (avg.), and difference (diff.) are given in percentages of successful classification experiments. The row labeled as size is for the corresponding sample size. The row labeled as #best is for the number of times the corresponding algorithm provided the best results.

	User2_Centre					User2_Walking					User2_Common				
	HMM	KNN	RF	NB	MLP	HMM	KNN	RF	NB	MLP	HMM	KNN	RF	NB	MLP
min. (%)	42.93	69.90	75.04	68.81	73.45	48.04	71.58	77.11	68.95	75.73	42.21	77.33	82.70	78.54	73.19
sample size	13	2	1	1	1	20	2	1	1	1	18	2	1	1	1
max. (%)	49.90	78.81	80.20	75.27	80.04	57.44	80.65	87.60	78.39	81.31	55.29	88.38	90.02	86.48	81.01
sample size	1	15	18	19	19	1	17	20	18	18	1	20	19	20	20
avg. (%)	45.11	75.85	78.26	72.82	77.91	51.02	77.36	84.02	74.76	79.36	44.97	83.55	87.44	83.14	78.06
diff. (%)	33.29	2.55	0.14	5.58	0.49	32.99	6.66	0.00	9.25	4.65	42.47	3.89	0.00	4.30	9.38
#best	0	0	12	0	8	0	0	20	0	0	0	0	20	0	0

**Table 5:** Summary of the data shown in Figure 9. The rows for minimum (min.), maximum (max.), average (avg.), and difference (diff.) are given in percentages of successful classification experiments. The row labeled as size is for the corresponding sample size. The row labeled as #best is for the number of times the corresponding algorithm provided the best results.

	User3_Centre					User3_Walking					User3_Common				
	HMM	KNN	RF	NB	MLP	HMM	KNN	RF	NB	MLP	HMM	KNN	RF	NB	MLP
min. (%)	71.19	14.77	63.28	58.98	24.50	41.80	35.93	77.71	51.70	58.20	66.18	47.24	71.09	62.64	59.67
sample size	1	19	1	1	19	1	2	1	1	1	1	2	1	1	2
max. (%)	84.79	21.00	74.59	63.56	26.67	83.18	48.10	85.57	59.28	65.43	87.83	64.26	79.31	72.37	68.47
sample size	19	5	20	4	1	20	19	19	19	15	15	17	19	17	20
avg. (%)	79.79	17.29	71.56	61.90	25.64	58.76	45.05	83.30	56.54	62.82	80.01	59.64	77.16	69.11	65.32
diff. (%)	0.00	62.50	8.23	17.88	54.15	24.54	38.25	0.00	26.76	20.48	1.05	21.42	3.90	11.95	15.74
#best	20	0	0	0	0	0	0	20	0	0	12	0	8	0	0

**Table 6:** Summary of the data shown in Figure 10. The rows for minimum (min.), maximum (max.), average (avg.), and difference (diff.) are given in percentages of successful classification experiments. The row labeled as size is for the corresponding sample size. The row labeled as #best is for the number of times the corresponding algorithm provided the best results.

	User1_All					User2_All					User3_All				
	HMM	KNN	RF	NB	MLP	HMM	KNN	RF	NB	MLP	HMM	KNN	RF	NB	MLP
min. (%)	75.20	69.52	76.14	46.69	74.13	72.86	83.79	86.71	70.57	80.08	66.18	47.24	71.09	62.64	59.67
sample size	1	2	1	2	1	18	2	1	1	1	1	2	1	1	2
max. (%)	82.12	82.49	82.60	52.03	82.58	75.30	93.41	92.46	78.77	88.16	87.83	64.26	79.31	72.37	68.47
sample size	14	20	20	19	19	7	20	19	20	20	15	17	19	17	20
avg. (%)	79.30	77.76	80.66	49.82	79.81	74.47	89.88	90.38	75.39	85.11	80.01	59.64	77.16	69.11	65.32
diff. (%)	1.49	3.03	0.13	30.97	0.98	16.16	0.75	0.25	15.24	5.52	1.05	21.42	3.90	11.95	15.74
best	5	0	14	0	1	0	10	10	0	0	12	0	8	0	0

**Table 7:** Results of z-test for comparison of the proposed method (HMM) against the other methods. NS stands for not significant.

Method	z-Value	Significance
KNN	0.0	NS
RF	−4.174	*p* < 0.01
NV	0.149	NS
MLP	−1.192	NS

**Table 8:** Mean computational time (miliseconds) and the mean squared for classifying a measure of five consecutive samples for all algorithms. The database used was user2_centre. Each experiment was repeated 100 times.

HMM	KNN	RF	NB	MLP
0.562±0.012	0.426±0.047	0.111±0.005	0.378±0.007	0.166±0.001

## Data Availability

Publicly available datasets were analyzed in this study. This data can be found here: http://www3.uji.es/~belfern/Research/Databases/wifi_databases.zip (Accessed: 29 March 2021).
